# Moderate Exercise Modulates Tumor Metabolism of Triple-Negative Breast Cancer

**DOI:** 10.3390/cells9030628

**Published:** 2020-03-05

**Authors:** Anderson Vulczak, Anderson de Oliveira Souza, Gustavo Duarte Ferrari, Ana Elisa Caleiro Seixas Azzolini, Gabriela Pereira-da-Silva, Luciane Carla Alberici

**Affiliations:** 1 Department of Biomolecular Sciences, School of Pharmaceutical Sciences of Ribeirão Preto, University of Sao Paulo, Ribeirão Preto, SP 14040-903, Brazil; andersonosouza@usp.br (A.d.O.S.); gustavoduartef@usp.br (G.D.F.); anael@usp.br (A.E.C.S.A.); 2 Department of Maternal and Child Nursing and Public Health, School of Nurse of Ribeirão Preto, University of Sao Paulo, Ribeirão Preto, SP 14040-902, Brazil; gbisson@eerp.usp.br

**Keywords:** mitochondria, OXPHOS, training, TNBC

## Abstract

Triple-negative breast cancer (TNBC) stands out for its aggressiveness and accelerated rate of proliferation. Evidence shows that exercise may exert antitumorigenic effects, but the biochemical mechanisms underlying them remain unclear. Our objective was to evaluate the ability of exercise to modulate tumor growth and energy metabolism in an experimental TNBC model. Female BALB/c mice were sedentary or trained for 12 weeks and inoculated with 1 × 10^4^ 4T1 cells in the eighth week. Analyzes of macronutrient oxidation, mitochondrial respiration, and expression of genes related to cell metabolism were performed. The results showed that the trained group had a smaller tumor mass and the mitochondria in the tumors presented lower respiratory rates in the state of maximum electron transport capacity. Additionally, the tumors of the exercised group showed a higher expression of genes related to tumor suppressors, while the genes linked with cellular growth were similar between groups. Furthermore, the training modulated the corporal macronutrient oxidation to almost exclusive carbohydrate oxidation, while the sedentary condition metabolized both carbohydrate and lipids. Therefore, the exercise reduced tumor growth, with an impact on mitochondrial and macronutrient metabolism. Our results shed light on the understanding of the antitumorigenic effects of physical exercise, particularly regarding the metabolic transformations in TNBC.

## 1. Introduction

Breast cancer ranks first among all cancers and the estimated incidence in 2018 was over two million new cases globally [1]. Triple-negative breast cancer (TNBC) represents 15%–20% of all breast cancers [2,3], and is characterized by an accelerated rate of proliferation, large tumor size, and highest risk of distant metastases [4]. These characteristics contribute to the poor prognosis and overall reduction in survival of patients with TNBC compared to other subtypes of breast cancer [3,4]. 

Although treatment strategies differ according to breast tumor subtype, to date no specific therapy for TNBC is available in clinical practice [3]. Despite breast cancer being a heterogeneous disease at the molecular level [5], some mutations are often found, for example, an increase in gene expression of the PTEN-Akt-mTOR pathway, linked to growth and cellular proliferation [5,6]. Also, mutations in TP53 reduced the expression of the protein-coupled cell cycle control [6]. Corroborating this, mutations in TP53 are closely associated with invasive breast cancer [7]. In addition to changes in gene expression related to cell cycle control, cell growth, and proliferation, tumor cells are also able to modulate gene expression related to energy metabolism control [8,9].

Thus, the tumoral metabolic alterations are suggested to confer adaptive advantages to cancer cells [9,10]. There is evidence that, while some tumors have high levels of oxidative phosphorylation (OXPHOS), others are relatively glycolytic and still maintain mitochondrial respiration and other functions [11,12,13]. In this regard, human TNBC cell line MDA-MB-468 presents higher oxygen consumption rates, whereas the HME1 subtype is more glycolytic [14]. In the murine experimental model of TNBC [15], the 4T1 cell line is dependent on functional mitochondria [16,17]. Therefore, metabolic changes that may favor tumor development are phenotype-dependent.

Nevertheless, evidence has indicated that delayed tumor adaptations can be achieved through physical exercise [18,19]. In this regard, some of the mechanisms proposed for the beneficial effects of physical exercise in cancers include the availability of substrates that affect tumor metabolism, besides hormonal changes, immune response modulation, and changes in tumor vascularization [18]. Although studies have reported an antitumor role of physical exercise in breast cancer [20,21,22], investigations of its effects on TNBC are still scarce and heterogeneous in the experimental model [22,23,24,25], especially regarding metastasis [26]. Therefore, whether exercise could modulate cancer metabolism and affect tumor development is still unclear. Hence, in this study, we investigated the effect of moderate aerobic physical exercise on energy metabolism in an experimental TNBC model.

## 2. Materials and Methods

### 2.1. Animals

All experiments were approved by the Animal Care and Use Committee of the School of Pharmaceutical Sciences of Ribeirão Preto, USP (Protocols no. 16.1.791.60.9). Female BALB/c (9 weeks old) mice were obtained from the University of São Paulo (USP) Campus of Ribeirão Preto. The animals were maintained at the vivarium of the Department of Physics and Chemistry, School of Pharmaceutical Sciences of Ribeirão Preto, USP, under an ambient temperature of 20 ± 2 °C, in a 12 h light/dark cycle with free access to chow and drinking water.

### 2.2. Animals and Exercise Protocols

**Figure 1:**
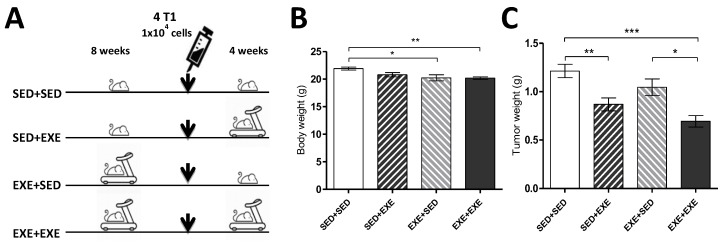
Physical exercises reduce body weight and tumor mass in the triple-negative breast cancer (TNBC) experimental model (4T1 cells). (**A**) Experimental design. (**B**) Body weight. (**C**) Tumor weight. One-way ANOVA with Bonferroni post hoc test. SED + SED *n* = 9; SED + EXE *n* = 8; EXE + SED *n* = 6; EXE + EXE *n* = 8. Means ± SEM. *p < 0.05; **p < 0.01; ***p < 0.001.

Female BALB/c mice performed forced moderate aerobic exercise on treadmill running (AVS™, Brasília, Brazil), 0% grade, 18 m/min for 30 min, five times per week, for 12 weeks. Prior to the exercise protocol, the mice performed 1 week of adaptation to treadmill running, with progressive time (10–15 min) and speed (8–12 m/min). The animals were divided into four groups, with sedentary (SED) and exercise (EXE) conditions all the time, while two other groups alternated between SED and EXE. The SED + SED (*n* = 9) group did not perform the exercise protocol, while the EXE + EXE (*n* = 9) group performed physical exercise throughout the experiment. The SED + EXE (*n* = 9) group performed exercise only after the induction of tumor cells, inversely to the EXE + SED (*n* = 9) group that performed treadmill exercises only until induction of tumor cells. After 8 weeks, all animals were injected orthotopically into the fourth-right dorsal mammary fat pad with 4T1 cells (1 × 10^4^) in suspension (Figure 1A). The animals that had no visual tumors after seven days were retired from the experiment (i.e., one animal of group SED + EXE, EXE + SED, and EXE + EXE, respectively). Two animals of the EXE + SED group died in the last day. Indirect calorimetry analysis was performed 24 h before tumor induction and 48 h before euthanasia. After 12 weeks, all mice were euthanized by cervical dislocation and tumor tissue was removed and weighted, and one part (~100 mg) was immediately separated for respirometry analysis and the remaining were stored at −80 °C.

### 2.3. Culture Cells

The murine mammary tumor cell line 4T1 was obtained from the American Type Culture Collection (ATCC). Cells were grown in 5% CO_2_ at 37 °C, with culture medium RPMI-1640™, supplemented with 10% fetal bovine serum, 1% penicillin-streptomycin (Sigma-Aldrich, St. Luis, MO, USA), and harvested by trypsinization at 70%–80% confluence in log phase growth on the day of tumor injection. The animals were injected orthotopically into the fourth-right dorsal mammary fat pad with 4T1 cells (1 × 10^4^) in suspension. Tumor size was measured from day 10 postinoculation of tumor cells using a caliper and was calculated according to the following formula: V = (DxL2)/2 (D = larger tumor diameter (mm); L = smaller tumor diameter (mm)). Tumor volume and body weight were measured twice weekly and animals were monitored continuously for the entire duration of exercise.

### 2.4. Indirect Calorimetry

The analysis of the body metabolism of the animals was performed by indirect calorimetry using the Oxylet Physiocage™ (PanLab, Barcelona, Spain) equipment. The experiment was carried in four metabolic boxes, with 04 animals/box. When more than two groups were analyzed, the animals from each group were divided into two subgroups, for later analysis, in order to carry out the analyzes concomitantly in all groups. The period of exposure in the metabolic box was 36–48 h, of which the final 24 h were used, corresponding to a complete light/dark cycle, for analysis of metabolic parameters. The first hours were avoided, when the animals were more active, adjusting to a new environment. The analyzed variables were the volume of oxygen consumed (VO_2_); the volume of carbon dioxide produced (VCO_2_); and the respiratory exchange ratio (RER) calculated by the relation [VCO_2_/VO_2_] used for the estimation of macronutrient oxidation.

### 2.5. Respirometry

Biopsies of tumors (35 mg) were chopped into 1 mm cubes and placed in ice-cold BIOPS solution containing saponin (0.01%) for plasma membrane permeabilization for 20 min. Biopsies (duplicates) were incubated in 2.1 mL air-saturated MiR05 (0.5 mM EGTA, 3 mM MgCl_2_, 60 mM K-lactobionate, 20 mM taurine, 10 mM KH_2_PO_4_, 20 mM HEPES, 110 mM sucrose, 1 g/L albumin, pH 7.1) at 37 °C, with 300 rpm stirring, and the respiratory rates were monitored (Oxygraph-2k, Oroboros, Innsbruk, Austria). The respiratory states were determined as follows: NADH-linked in the presence of exogenous substrates (substrates, S state: 9 mM glutamate, 5 mM malate); phosphorylation, after addition of adenosine diphosphate (phosphorylating or P state: 1 mM ADP); nonphosphorylating, after ATP synthase inhibition by oligomycin (nonphosphorylating, leak or L state: 1 µg/mL); noncoupled, in the presence of the mitochondrial uncoupler carbonyl cyanide m-chlorophenyl hydrazone (uncoupled or E state: CCCP, 1 or 2 µM); and residual (ROx), after complex III inhibition by antimycin A (3 µM AA) [27] and subtracted from every respiratory state. After the determination of respiratory rates, the reaction mixture was used for protein quantification [28]. The integrity of the mitochondrial inner membrane was checked using a cytochrome c test [29].

### 2.6. Citrate Synthase Activity Assay and Lactate Quantification

After tissue (100 mg) homogenization in RIPA buffer (0.75 M NaCl, 0.5 % SDS, 0.25 M Tris, 5% Triton X-100, 100 mM EDTA supplemented with 100 mM orthovanadate, 100 mM sodium pyrophosphate, 100 mM PMSF, 1% leupeptin) proteins were quantified by the Bradford method. For the analysis of citrate synthase activity, duplicates of 10 µg of protein were incubated with reaction medium (50 mM Tris-HCl, 100 μM 5,5’-di-thiobis-(2-nitrobenzoic acid) (DTNB), 0.25% Triton X-100, pH 8.0, supplemented with 50 μM acetyl-CoA) at 37 °C for 10 min. The reaction was started with the addition 250 μM oxaloacetate [30]. The absorbance was measured every 4 s for 5 min. The lactate quantification was performed with enzymatic lactate assay Labtest^TM^ using 10 µL of homogenate tumor tissue in duplicates, according to the manufacturer’s guidelines and subsequently quantification was normalized by protein content.

### 2.7. qPCR Analysis

Total RNA was isolated from frozen tumor samples by tissue homogenization and RNA extraction using the TRIzol method (Invitrogen). Quality and quantity of the isolated total RNA were determined by a Nanodrop spectrophotometer (Thermo Scientific, Waltham, MA, USA). The total RNA (500 ng) was reversely transcribed into complementary DNA (cDNA). All amplifications were carried out in triplicate out to a final volume of 12 µL with 1 µL cDNA and monitored in real time using the StepOne^TM^ Real-Time PCR System (Applied Biosystems) with Master Mix as a fluorescence marker (GoTaq^TM^ qPCR Systems, Promega) using sequence-specific primers (Table 1). Quantification was performed by normalizing to standard curves for each gene. Target genes were normalized to the housekeeping GAPDH or β-Actin gene, and the fold change in gene expression was determined using the ΔΔCt method [31].

### 2.8. Statistical Analysis

All statistical analyses were performed using Graph Prism™ 5.0 (GraphPad, La Jolla, CA, USA). Variables were compared with Student’s *t*-test, two-tailed for two groups, and ANOVA with four groups. For analysis, a *p*-value < 0.05 was considered significant.

## 3. Results

### 3.1. Moderate Aerobic Exercise Reduces Body Weight and Tumor Mass

The exercise training resulted in lower body weights in animals of EXE + SED and EXE + EXE groups, compared to SED + SED before the euthanasia (Figure 1B). Four weeks after tumor cell injection, the average tumor weight was significantly lower in SED + EXE and EXE + EXE groups compared to SED + SED. In addition, tumor weight in EXE + EXE was also lower than in the EXE + SED group (Figure 1C).

### 3.2. Exercise Training During the Tumorigenic Process Increases Body Carbohydrate Oxidation in the TNBC Experimental Model

The evaluation of the RER for 24 h showed that before tumor inoculation (RER pretumor), sedentary groups (SED + SED and SED + EXE), presented a value close to 1.0, characterized by predominant use of carbohydrates as an energy substrate. Conversely, groups that performed physical exercise (EXE + SED and EXE + EXE) had lower RER pretumor values, which indicate the use of both carbohydrates and lipids as energy substrates. Four weeks after tumor induction (RER post tumor), the SED + SED group presented a reduction of the RER, demonstrating that a sedentary condition during tumor growth (SED + SED and EXE + SED) modulated the oxidation of carbohydrates and lipids, while the groups that performed exercises during the tumorigenic process, SED + EXE and EXE + EXE, had an RER close to 1.0, demonstrating the predominant carbohydrate oxidation in this period [32] (Table 2).

### 3.3. Moderate Exercise Reduces Mitochondrial Respiratory Capacity in the TNBC Experimental Model

**Figure 2:**
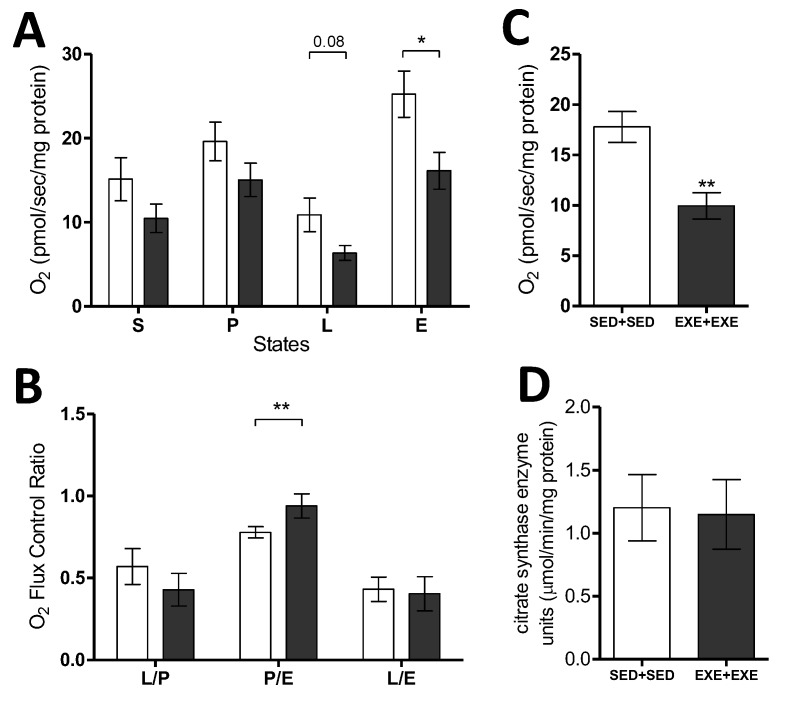
Physical exercise reduces mitochondrial capacity in tumor tissue of TNBC (4T1 cells). (**A**) Oxygen consumption rates in states with substrates glutamate, malate, and pyruvate (S); ADP (phosphorylating, P); oligomycin (nonphosphorylating, leak, L); CCCP (uncoupled, E). SED + SED *n* = 9; EXE + EXE *n* = 7. (**B**) Flux Control Ratios: leak as a fraction of phosphorylation (L/P); phosphorylation as a fraction of the maximum electron transport capacity (P/E); leak as a fraction of the maximum electron transport capacity (L/E). SED + SED *n* = 9; EXE + EXE *n* = 7. (**C**) Residual oxygen consumption (ROx) after antimycin A. SED + SED *n* = 9; EXE + EXE *n* = 7. (**D**) Activity of the citrate synthase enzyme. SED + SED *n* = 9; EXE + EXE *n* = 7. White bars = SED + SED, gray bars = EXE + EXE. Unpaired *t*-test, Means ± SEM. *p < 0.05; **p < 0.01.

Considering the prominent differences between the SED + SED and EXE + EXE groups regarding tumor growth and body energy metabolism, these two groups were chosen for analysis of tumor bioenergetics. The O_2_ consumption monitoring showed that the tumor tissue of trained animals presented lower respiratory rates in all respiratory states, and a statistically significant reduction in the state of maximum electron transport capacity (E), demonstrating a reduced mitochondrial respiratory capacity. The nonphosphorylating respiratory state (L) tended to be reduced in the trained group, however, no statistical difference was observed (*p* = 0.08) (Figure 2A). The analyses of Flux Control Ratios did not show differences in the L/E and L/*p* between the groups, while the phosphorylation control (P/E) was significantly higher in the EXE-EXE group, demonstrating a mitochondrial oxidative phosphorylation capacity closer to their maximum respiratory limit when compared to the sedentary group (Figure 2B). Additionally, the oxygen consumption not related to the mitochondrial activity (ROx) was lower in the tumors of the trained group, indicating a lower activity of cellular oxidases (Figure 2C). To verify if the lower mitochondrial respiratory capacity in the tumor tissue of the trained animals was related to mitochondrial content, the activity of citrate synthase (CS), a classical biomarker of mitochondrial density, was measured. The results revealed the similarity between the sedentary and trained groups to CS (Figure 2D), indicating that the tumors of EXE-EXE group had a lower mitochondrial capacity independent of mitochondrial density, compared to the tumors of sedentary group.

### 3.4. Exercise Training Modulates the mRNA Expression Related to the Control of Tumoral Process

**Figure 3:**
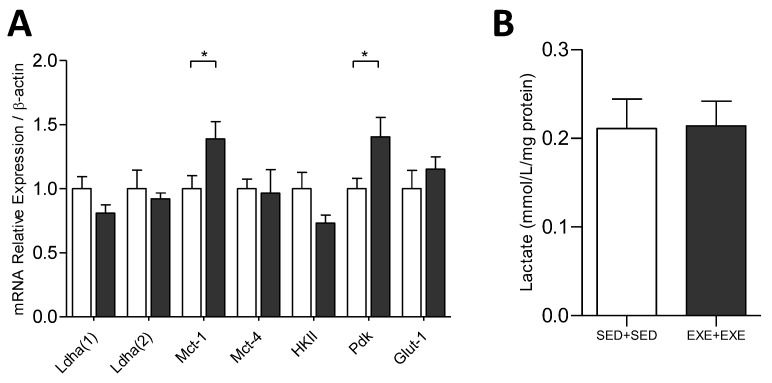
Physical exercise modulates the expression of genes related to glycolytic metabolism but not lactate concentration in tumor tissue of TNBC (4T1 cells). (**A**) Relative expression of genes related to glucose metabolism. Ldha (1), lactate dehydrogenase A isoform 1; †Ldha (2), lactate dehydrogenase A isoform 2; Mct-1, monocarboxylate transporter 1; †Mct-4, monocarboxylate transporter 4; HKII, hexokinase II; Pdk, pyruvate dehydrogenase kinase; Glut-1, glucose transporter type 1; β-actin, housekeeping. SED + SED *n* = 6; EXE + EXE *n* = 6. † group SED + SED *n* = 4; group EXE + EXE *n* = 4. (**B**) Lactate concentration in tumor tissue. SED + SED *n* = 7; EXE + EXE *n* = 7. White bars = SED + SED, black bars = EXE + EXE. Unpaired *t*-test, Means ± SEM. *p < 0.05.

The relative mRNA expression of the genes related to glucose metabolism Ldha, Mct, HKII, Pdk, and Glut 1 demonstrated an increase only in the expression of Mct-1 and Pdk in the tumors of the trained group. Mct-1 encodes a protein responsible for importing lactate from the extracellular medium [33], and Pdk-1 encodes a protein that inhibits pyruvate dehydrogenase and consequently alters mitochondrial function [8]. In addition, lactate content in tumor tissue was similar between sedentary and trained groups (Figure 3).

**Figure 4:**
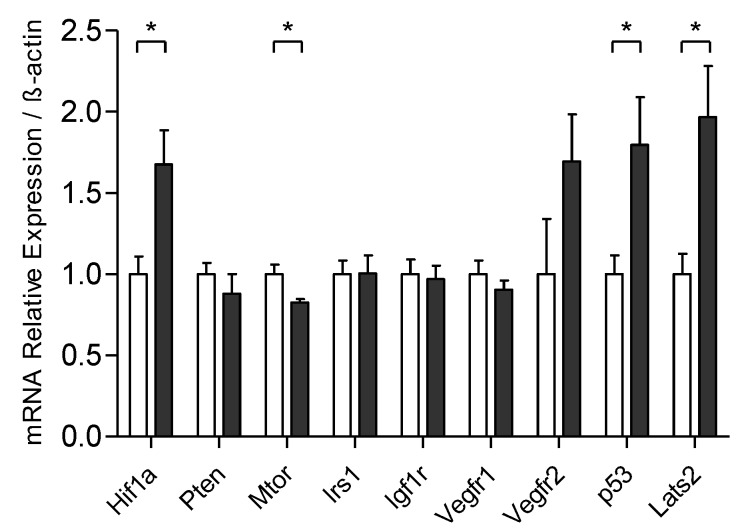
Exercise influences the expression of genes related to the control of tumor growth in TNBC (4T1 cells). Relative mRNA expression of Hif1a, hypoxia-inducible factors; Pten, phosphatase and tensin homolog; Mtor, mammalian target of rapamycin; Irs1, insulin receptor; Igfr1, insulin-like growth factor 1 receptor; Vegfr (1–2), vascular endothelial growth factor receptor; p53, tumor protein p53; Lats, large tumor suppressor; β-actin, housekeeping, SED + SED *n* = 4; EXE + EXE *n* = 4. White bars = SED + SED, gray bars = EXE + EXE. Unpaired Student *t*-test, Means ± SEM.*p < 0.05.

Despite upregulation in the gene related to hypoxia (Hif1a), no difference in Irs1, Igf1r, Vegf, and Pten relative expression (related with cellular growth) was observed between the groups, while Mtor mRNA levels decreased after training. However, the tumor suppressor gene expression of p53 and Lats2 was higher in the trained group (Figure 4).

## 4. Discussion

Investigations of the effects of physical exercise on experimental TNBC are still scarce and wield heterogeneous results [22,23,24,25,26]. Here we showed that moderate aerobic physical training, both 8 weeks before and 4 weeks after the inoculation of 4T1 cancer cells, reduces tumor growth. However, training only after tumor induction demonstrated anti-tumorigenic effects to a lesser extent. Interruption of training after inoculation of tumor cells favored tumor growth similarly to the full-time sedentary group. Corroborating our results, Bianco and colleagues [24] showed that forced swimming exercise reduced tumor volume in animals inoculated with 2 × 10^5^ 4T1 cells. In addition, voluntary physical exercise has been shown to reduce tumor growth rate after mice were inoculated with 5 × 10^4^ [22] or 1 × 10^4^ [23] 4T1 cells. Conversely, moderate physical exercise on the treadmill was not able to affect TNBC tumor growth in mice inoculated with 1 × 10^6^ 4T1 cells [34]. Furthermore, in three different subtypes (experimental models) of TNBC, animals that were submitted to aerobic exercise on treadmill exhibited different responses to the same training program [25], demonstrating that the anti-tumorigenic effect of physical exercise is not forthright. Therefore, tumor subtype, number of inoculated cells, and volume/intensity of training are variables that drive the anti-tumorigenic effects of physical exercise. Although many studies examine the effects of exercise on tumor development, the knowledge of how training exerts antitumoral effects is unclear.

To our knowledge, this is the first study on the impact of physical exercise on mitochondrial metabolism of TNBC. Our results demonstrated that the mitochondrial activity of tumor cells from exercised animals was lower in comparison to the tumor cells of sedentary animals, with a significant decrease in the electron transport chain capacity (E), demonstrating lower respiratory capacity independently of the mitochondrial content, measured by CS activity. We also observed a higher P/E ratio in the group of trained animals, indicating that almost total mitochondrial respiratory capacity was used by oxidative phosphorylation. Mitochondrial impairment is also reinforced by increased Hif1a, which positively regulates glycolytic genes [8], such as Pdk gene expression, which encodes a protein that inhibits the pyruvate dehydrogenase complex, which is the main source of supply for the Krebs cycle. The role of mitochondria in tumor metabolism remains controversial in cancer research [12,35]. Although increased glycolysis is a hallmark of tumor growth, investigations on tumor metabolism and the importance of OXPHOS in the microenvironment and tumor bioenergetics have been highlighted in the literature [10,12,35]. In the experimental model of TNBC with 4T1 cells, mitochondrial respiration has been considered fundamental for tumor growth and progression [16], as well as a xenograft model of TNBC, whose MDA231 cells are dependent on mitochondrial fatty acid oxidation [36]. A similar result was also reported in studies with a syngeneic experimental model with melanoma from B16 cells [37,38] and with pancreatic ductal adenocarcinoma, which demonstrated that tumor recurrence is dependent on oxidative phosphorylation for the survival of tumor cells [39]. In this regard, we observed that the sedentary group had higher oxidative phosphorylation capacity compared to the trained group. Correspondingly, the re-establishment of OXPHOS has been associated with tumor aggressiveness and, consequently, the appearance of metastasis in 4T1 cells [16], demonstrating the importance of mitochondrial energy metabolism for tumor development. Moreover, we observe a higher residual oxygen consumption (ROx) in the sedentary group, which occurs through the activity of cellular enzymes that consume oxygen and auto-oxidation reactions [27], and has been shown to favor the progression of breast cancer in animals inoculated with 4T1 cells [40,41].

The tumor mass was forty-three percent smaller in trained animals. Corroborating this, the trained group when compared to sedentary showed no gene expression differences linked to cell growth (i.e., Pten, Irs-1, Igf1r, Vegfr), combined with a slight reduction in Mtor relative expression in the trained group. Research on exercise and breast cancer that assess the expression of the genes of the tumor energy metabolism are scarce and quite heterogeneous, making data interpretation challenging. For example, Glass and colleagues [25] identified differences in two TNBC models: reduction of tumor volume through training showed reduced expression of Hif-1α and no change in Glut-1 expression in EO771 cells, however, in transgenic animals (C3 (1) SV40 Tag p16-luc) the training caused an increase in Hif-1α and a reduction in Glut-1 expression, with a consequent increase in tumor size. In this regard, in a xenographic experimental model with MDA-MB-231 cells, although tumor size remained unchanged, HIF-1 expression and tumor vascularization increased in the trained group [42]. In contrast, in the MC4-L2 experimental model, the training was associated with reduced angiogenesis and consequently decreased tumor size [43]. Another study with MC4-L2 cells reported a reduction in the expression of LDH-A and MCT-1 and increased LDH-B in trained animals [20]. Noteworthy, the heterogeneity of experimental models, particularly concerning the training protocols, hinders the understanding of the impact of physical exercise on tumor energetic metabolism.

Although mRNA analysis does not show direct binding to protein function, it is evident that the training modulates the gene regulation and can affect tumor growth. Interestingly, our results demonstrate that the training increased the relative expression of tumor suppressors, such as p53 and Lats2, suggesting that physical exercise can activate mechanisms of tumor suppression and delay tumor growth. A recent study that used the cross-talk model of cell culture and medium supplemented with the serum of exercised women, showed that MCF-7 cells, but not MDA-MB-231 cells, had a suppressive effect of the physical exercise, mediated by the Lats2 tumor suppressor [44]. In addition, the inactivation of p53, or a combined loss of p53 and Rb, in the mammary epithelium, results in mammary carcinomas that present recurrent LATS2 inactivation [45]. Besides, LATS2 and p53 interact through positive feedback to ensure cell differentiation or cellular senescence in response to stress in replication [46]. Furthermore, in response to mitotic stress, LATS2 binds to and inhibits MDM2 and consequently, p53 activation induces cell cycle arrest in the G1/S phase [46]. Therefore, one of the possibilities of tumor suppressor activation is related with energy metabolism, whose nutrients restriction can activate p53 and Lats2, causing cell cycle arrest [47].

Nevertheless, physical exercise has an important impact on RER, and consequently global modulation in macronutrient oxidation, both during physical exercise and the recovery period [48,49]. Corroborating this, we demonstrated through indirect calorimetry analysis that, whereas sedentary tumor-bearing animals had both lipid and carbohydrate oxidation, animals trained during tumor development growth used almost exclusively carbohydrates as the main energy source for maintaining a resting metabolic rate. In this regard, Goh and colleagues, using an experimental model with 4T1 cell inoculation, also showed an enhancement in carbohydrate oxidation and smaller tumor mass in the trained animals [23]. Moreover, in a colorectal xenografts model, the metabolomics analysis demonstrated that voluntary exercise affected carbon metabolism-coupled mitochondrial metabolism [50]. Therefore, despite the tumor growth affects, the corporal metabolism, the modulation in RER, and macronutrient oxidation from training seem to overlap with the tumor metabolism in the experimental 4T1 tumor model, which is dependent on mitochondrial metabolism and carbohydrate supply as the energy input. Interestingly, we found no differences in the tumors’ lactate content, and although lactate dehydrogenase A is necessary for tumor maintenance and progression [51,52], our mRNA analysis of the two Ldh-a transcript variant was also similar between the sedentary and trained group, despite the impairment of mitochondrial respiration in the tumors of trained animals. The Ldh-a transcript variant 2 uses an alternate 5′ exon that results in a distinct 5′ UTR and translation initiation at an alternat codon, resulting in a longer N-terminus compared to isoform 1. The assay of two transcript variants was made to confirm the data on Ldh-a gene expression. Conversely, physical exercise increased MCT-1 (lactate import) gene expression, which may indicate that the tumor might be consuming the lactate from the microenvironment. The mRNA expression provides information on the possible physiological changes, and further analysis of protein levels is necessary to confirm our results. Although we have no data on tumor multiplicity and metastatic potential, taken together our results demonstrated that moderate aerobic physical exercise reduces TNBC tumoral growth with effect on tumor metabolism, characterized by a reduction in mitochondrial respiratory capacity, an important factor for tumor growth in the experimental model using 4T1 cell inoculation in mice. Given our observations that the trained group showed increased gene expression of tumor suppressors sensitive to the availability of nutrients, besides modulation of the respiratory exchange ratio from carbohydrate oxidation, it is suggestive that the effect of exercise on growth delay in tumors may be linked to carbohydrate metabolism and mitochondrial function. Our study opens new perspectives for the identification of metabolic pathways sensitive to exercise, allowing a better understanding of its anti-tumorigenic effects.

## Figures and Tables

**Table 1:** Primers analyzed by qPCR.

Gene	Forward	Reverse
*Ldha iso1*	ATCGTGCACTAGCGGTCTCA	CCATCATCTCGCCCTTGAGT
*Ldha iso2*	AACTTGGCGCTCTACTTGCT	GGACTTTGAATCTTTTGAGACCTTG
*Mct1*	ACGCCGGAGTCTTTGGATTT	TGAGGCGGCCTAAAAGTGG
*Mct4*	GCACTTAAAGTCGCCCCCG	GAGGGCTGCTTTCACCAAGAAC
*HKII*	GGAGTCTTCGATCCCAGCCG	CTGGTCAACCTTCTGCACTTGG
*Pdk 1*	AAGCAGTTCCTGGACTTCGG	GGCTTTGGATATACCAACTTTGC
*Glut-1*	GTGACGATCTGAGCTACGGG	TCACCTTCTTGCTGCTGGG
*Hif1a*	TGAGTTCTGAACGTCGAAAAGA	TAGACCACCGGCATCCAGA
*Pten*	CAGGCTCCCAGACATGACA	GCTTTGAATCCAAAAACCTTACTAC
*Mtor*	TCACTTCCTGAACAGCGAGC	GTAGCGGATATCAGGGTCAGG
*Irs-1*	CCGATATGGTGATGAGGAGCTG	TGGCAATATTTGATGGGACATCT
*Igf1r*	GCACCAATGCTTCAGTCCCT	TTGGAGCAGTAGTTGTGCCG
*Vegfr 1*	GTGTCTATAGGTGCCGAGCC	CGGAAGAAGACCGCTTCAGT
*Vegfr 2*	GCATACCGCCTCTGTGACTT	AAATCGCCAGGCAAACCCAC
*p53*	TCCGAAGACTGGATGACTGC	GCTTCACTTGGGCCTTCAAA
*Lats 2*	AGCTGAAGGTGATCAACTGGG	CAGGCTGCTTTCGGATGTCA
*β Actina*	GATCAAGATCATTGCTCCTCCTG	AGGGTGTAAAACGCAGCTCA

**Table 2:** Evaluation of the respiratory exchange ratio (RER) by indirect calorimetry (24 h).

	(SED + SED)	(SED + EXE)	(EXE + SED)	(EXE + EXE)
*RER _pretumor_*	*0.98 ± 0.08*	*0.97 ± 0.11*	*0.90 ± 0.11 ^a,b^*	*0.89 ± 0.10 ^a,b^*
*RER _post-tumor_*	*0.92 ± 0.12^†^*	*1.00 ± 0.07 ^a,d,‡^*	*0.88 ± 0.14 ^b,c^*	*0.98 ± 0.09 ^a,d,‡^*

RER pretumor: Preinoculation respiratory exchange ratio of tumor cells; RER post-tumor: Respiratory exchange ratio after 4 weeks of tumor inoculation, 36 h before sacrifice; SED + SED *n* = 9; SED + EXE *n* = 8; EXE + SED *n* = 6; EXE + EXE *n* = 8; one-way ANOVA with Bonferroni post hoc test (*a*: ****p < 0.0001 vs. SED + SED; *b*: ****p < 0.0001 vs. SED + EXE; *c*: *p < 0.05 vs. SED + SED; *d*: ****p < 0.0001 vs. EXE + SED). Paired *t*-test (pretumor vs. post-tumor), mean ± standard deviation (†: ***p < 0.001; ‡: ****p < 0.0001).
